# Toward incorporating genetic risk scores into symptom networks of psychosis

**DOI:** 10.1017/S003329171900045X

**Published:** 2020-03

**Authors:** Adela-Maria Isvoranu, Sinan Guloksuz, Sacha Epskamp, Jim van Os, Denny Borsboom, GROUP Investigators

**Affiliations:** 1 Department of Psychology, Psychological Methods, University of Amsterdam, Amsterdam, The Netherlands; 2 Department of Psychiatry and Neuropsychology, School of Mental Health and Neuroscience, Maastricht University Medical Center, Maastricht, The Netherlands; 3 Department of Psychiatry, Yale School of Medicine, New Haven, CT, USA; 4 Utrecht University Medical Centre, Utrecht, The Netherlands

**Keywords:** GWAS, network analysis, polygenic risk score, psychosis, schizophrenia

## Abstract

**Background:**

Psychosis spectrum disorder is a heterogeneous, multifactorial clinical phenotype, known to have a high heritability, only a minor portion of which can be explained by molecular measures of genetic variation. This study proposes that the identification of genetic variation underlying psychotic disorder may have suffered due to issues in the psychometric conceptualization of the phenotype. Here we aim to open a new line of research into the genetics of mental disorders by explicitly incorporating genes into symptom networks. Specifically, we investigate whether links between a polygenic risk score (PRS) for schizophrenia and measures of psychosis proneness can be identified in a network model.

**Methods:**

We analyzed data from *n* = 2180 subjects (controls, patients diagnosed with a non-affective psychotic disorder, and the first-degree relatives of the patients). A network structure was computed to examine associations between the 42 symptoms of the Community Assessment of Psychic Experiences (CAPE) and the PRS for schizophrenia.

**Results:**

The resulting network shows that the PRS is directly connected to the spectrum of positive and depressive symptoms, with the items *conspiracy* and *no future* being more often located on predictive pathways from PRS to other symptoms.

**Conclusions:**

To our knowledge, the current exploratory study provides a first application of the network framework to the field of behavior genetics research. This allows for a novel outlook on the investigation of the relations between genome-wide association study-based PRSs and symptoms of mental disorders, by focusing on the dependencies among variables.

## Introduction

Psychosis spectrum disorder is a potentially severe, heterogeneous, and multifactorial disorder (Keshavan *et al*., 2011; Guloksuz and van Os, 2017). Although twin and family studies indicate substantial heritability, few genetic variants are consistently linked to psychotic disorder (Pardiñas *et al*., 2018). The difficulty in identifying genes specific to psychosis has been explained in several ways (Sullivan, 2008). One possibility that has received scant attention is that the conceptualization of the phenotype may be suboptimal: typically, genetic studies use *symptom counts* (e.g. total scores defined on questionnaire data) or case–control designs that define cases and controls as polythetic functions of symptom data (i.e. the definition of cases corresponds to many distinct symptom profiles), thus defining the phenotype in a highly simplified fashion. Essentially, these approaches assume that such compound scores are estimates of a single underlying dimension (e.g. a *liability spectrum*) that partly stands under genetic control (Franić *et al*., 2013). Additionally, genetic research uses clear-cut diagnostic categories, even though dominant liability spectrum theories are more consistent with a spectrum of related phenotypes (van Os and Linscott, 2012), which feature no single set of necessary and sufficient properties (e.g. a *disease entity* or *common pathogenic pathway*).

Several recent papers have argued that this conceptualization of mental disorders may be too simplistic. Instead of reflecting a single disease entity, pathogenic pathway, or unidimensional liability spectrum, disorders may result from causal interactions between symptoms (e.g. delusions→paranoia→social isolation), which constitute a network of symptoms and other components (Cramer *et al*., 2010; Borsboom and Cramer, 2013; Borsboom, 2017). Within this *network conceptualization*, the focus shifts from investigating mental disorders – such as schizophrenia – as *disease entities* to investigating interactions between symptoms (Borsboom and Cramer, 2013; Borsboom, 2017). Thus, mental disorders do not result from one central dysfunction (e.g. a brain dysfunction) that causally produces symptoms, but from a complex interplay between symptoms, psychological, biological, and sociological components (Epskamp, 2017; Isvoranu *et al*., 2019).

For psychosis spectrum disorders, which feature a clear genetic signal, this raises the question of how genetic factors could relate to symptom–symptom interactions in a network structure. In our view, genetic risk could plausibly influence symptom networks in two ways. First, genetic risk could act as a *direct main effect* on symptoms. In this case, genetic risk would be conductive to the *liability* to develop *certain symptoms* of schizophrenia; this liability could be thought of in terms of genetic make-up laying the foundations for symptoms to develop. For instance, one's genetic make-up could have an effect on the likelihood that the symptom ‘hallucinations’ occurs, so that higher genetic risk influences the liability of developing the symptom, which in turn would activate other neighboring symptoms in a network. Second, genetic risk could function as a *moderator* of the network structure: genetic risk may involve genetic factors that increase the *strength* of a causal connection between symptoms. For instance, genetics may predispose a person to experience (more) anxiety in response to hallucinations. In this scenario, genetic factors control part of the *structure* of a network, such that a genetic risk would be expressed as the likelihood that one symptom activates another. Analyses that would optimally represent the moderation hypothesis are not yet fully developed, and therefore the current manuscript is focused on the first approach.

To the extent that the above scenarios are plausible, current genetic studies using total scores defined by these symptoms may rest on an inadequate definition of the phenotype. In particular, genetic markers or polygenic risk scores (PRSs) may be more profitably analyzed in relation to a network phenotype, instead of to a total score or case–control status. The current paper aims to introduce methodology suited to incorporate genetic risk scores – in particular, genome-wide association study (GWAS)-based PRSs for schizophrenia – into symptom networks, thus opening new lines of research into the genetics of mental disorders. PRS is commonly used to summarize genetic effects and represents the sum of trait-associated alleles across many genetic loci, weighted by effect sizes estimated from GWAS (Euesden *et al*., 2015). PRS is generally used to identify genetic basis of phenotypes and to construct risk prediction models (Dudbridge, 2013). Specifically, here we investigate whether links between PRS for schizophrenia and measures of psychosis proneness can be identified in a network model, by including the PRS as a variable in the symptom network for psychosis spectrum disorder.

## Method

### Participants

The sample analyzed was part of the longitudinal observational cohort study ‘Genetic Risk and Outcome of Psychosis Project’ (GROUP), release database 7.0 (Korver *et al*., 2012). At baseline, the GROUP sample consisted of 1119 patients diagnosed with a non-affective psychotic disorder, 1059 siblings of these patients, 920 parents, and 586 unrelated healthy controls. The patients were recruited from mental health care institutions across The Netherlands and Belgium and the control subjects were recruited through random mailing. Inclusion criteria for patients were age 16–50 years, Diagnostic and Statistical Manual of Mental Disorders (American Psychiatric Association, 2000) criteria for a non-affective psychotic disorder, maximum duration of illness of 10 years, and estimated level of intelligence quotient (IQ) above 70. For full details on the GROUP sample please refer to the cited paper (Korver *et al*., 2012).

The current study used baseline data, restricted to the European white ethnic group, as there is evidence for a differential impact of PRS in different ethnic groups (Marden *et al*., 2014; van Os *et al*., 2017). Only subjects for which a PRS was available were included in the analysis. Overall, a baseline sample of *n* = 2180 individuals was analyzed here (patients, siblings, parents, and controls).

### Symptomatology

We used baseline data from the Community Assessment of Psychic Experiences (CAPE; Konings *et al*., 2006), a self-report measure of lifetime psychotic experiences. All items, measuring frequency of positive (20 items), negative (14 items), and depressive symptoms (8 items) were included in the analyses and were scored on a 4-point Likert scale.

### Genotyping, imputation, and PRS

A standard procedure for genotyping, imputation, and PRS was used. For a detailed procedure refer to online Supplementary Appendix S1.

### Network construction

We fitted a Gaussian graphical model (GGM; Lauritzen and Wermuth, 1989) to the data (i.e. an undirected network). All items of the CAPE and the PRS were represented as *nodes*. An *edge* between any two nodes indicates a partial correlation between the two variables, after conditioning on all other variables in the dataset. To account for the ordinal nature of the CAPE data, we used Spearman correlations when estimating the network structure, as recommended by Epskamp and Fried (2018). Due to the high sample size, we used unregularized model selection rather than regularization techniques commonly used in estimating GGMs^1^ (Williams and Rast, 2018). All analyses were performed using R-statistical software, version 3.4.3 (R Development Core Team, 2014). The networks were constructed and visualized using the R-package *qgraph* version 1.4.5 (Epskamp *et al*., 2012).

Blue (red) edges in the network indicate positive (negative) partial correlations, and the wider and more saturated the edge, the stronger the partial correlation (Epskamp *et al*., 2012). Because the current paper specifically focuses on edges between the PRS and symptoms, edges representing associations between PRS and symptoms have been manually unfaded.^2^ For constructing the layout of the network, we used a manually specified layout where the PRS is positioned at the center of the network, while allowing the rest of the nodes in the network to cluster based on their associations.

To display how the connectivity of edges is related across genetic and symptomatic levels of analysis, we computed a *predictive path diagram* (Fig. 2). A *predictive path diagram* takes one node (in our case PRS) as a source node and then lists (i) the nodes that have an edge with PRS; (ii) the nodes to which the nodes that have an edge with PRS are connected; (iii) the nodes to which the latter are connected – and so forth. Within the so constructed diagram, the *shortest predictive pathway* can be defined as the pathway that results from traveling the edges that, in each step, maximize the quality of the prediction of the target node from the source node, while controlling for all other variables in the network. To investigate which nodes more often lie on the shortest predictive pathways from PRS to other nodes we computed node-specific predictive betweenness as a centrality measure (Fig. 3). Because betweenness is generally not a very stable centrality measure (Epskamp *et al*., 2018), we used both nonparametric and case-drop bootstraps to investigate the extent of its variability (Epskamp *et al*., 2017).

### Network stability

As recommended in the literature (Borsboom *et al*., 2018), to investigate robustness and replicability of results we performed accuracy and stability checks using the R package *bootnet* (Epskamp *et al*., 2017). In addition, we included a stability analysis to investigate the average node-specific predictive betweenness under case-dropping. For these results we refer the reader to online Supplementary Appendix S2.

## Results

The study sample at baseline consisted of 2180 subjects (335 control subjects, 640 siblings, 630 parents, and 575 patients), of which 47% female and 53% male. Within the patient sample, the main diagnosis was *schizophrenia*, *paranoid type* (53%), followed by *psychotic disorder NOS* (11%), *schizoaffective disorder* (10%) and *schizophreniform disorder* (6%). The mean age of the subjects was 35.97 (14.61) years and the mean IQ was 103.48 (16.17). In the current analyses a GWAS *p* value threshold of 0.05 was used. In total, 194 665 single-nucleotide polymorphisms (SNPs) were included in the PRS.

### Network analysis

**Fig. 1.**
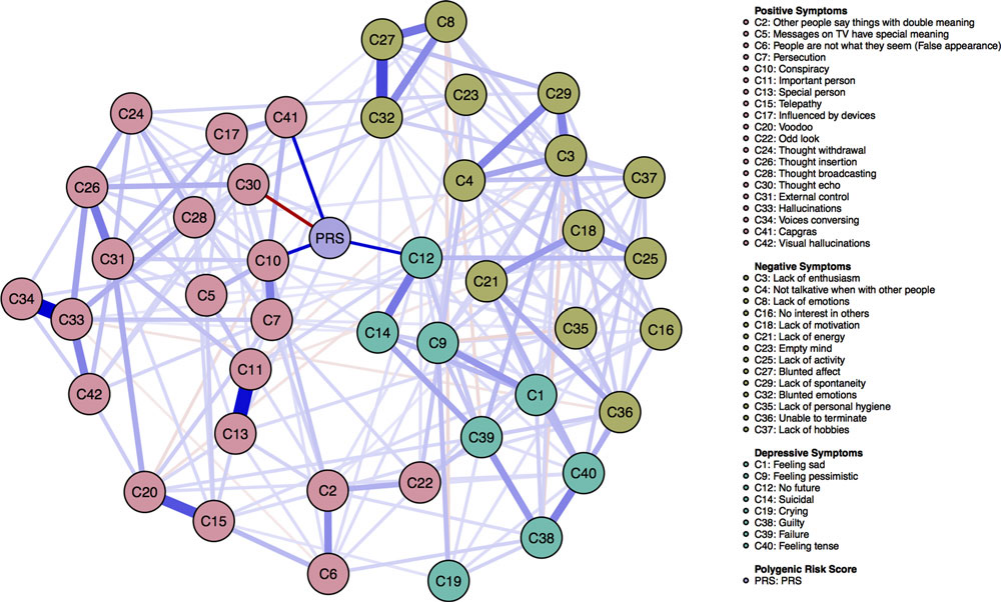
Network of the 42 CAPE (Konings *et al*., 2006) symptoms and the PRS for psychosis (*n* = 2180). Blue (red) lines represent positive (negative) associations between variables and the wider and more saturated the edge, the stronger the association (Epskamp *et al*., 2012). Please note that since the focus of the paper was to investigate the relations between the PRS and symptoms, the edges between the PRS and symptoms have been manually un-faded, while the edges between the other nodes in the network retain transparency. Symptom groups are differentiated by color.

Figure 1 presents the network depicting positive, negative, and depressive symptoms of the CAPE, as well as the PRS. Results show that the PRS is directly connected to several individual symptoms of the CAPE, but especially related to the spectrum of positive psychotic symptoms. Network analysis identified relations between the PRS and the positive psychotic symptoms *C10* (conspiracy), *C30* (thought echo), and *C41* (Capgras). In addition, we identified positive relations between the PRS and the depressive symptom *C12* (no future).^3^ Stability analyses show that the network and identified edges are generally stable (see online Supplementary Appendix S2).

**Fig. 2.**
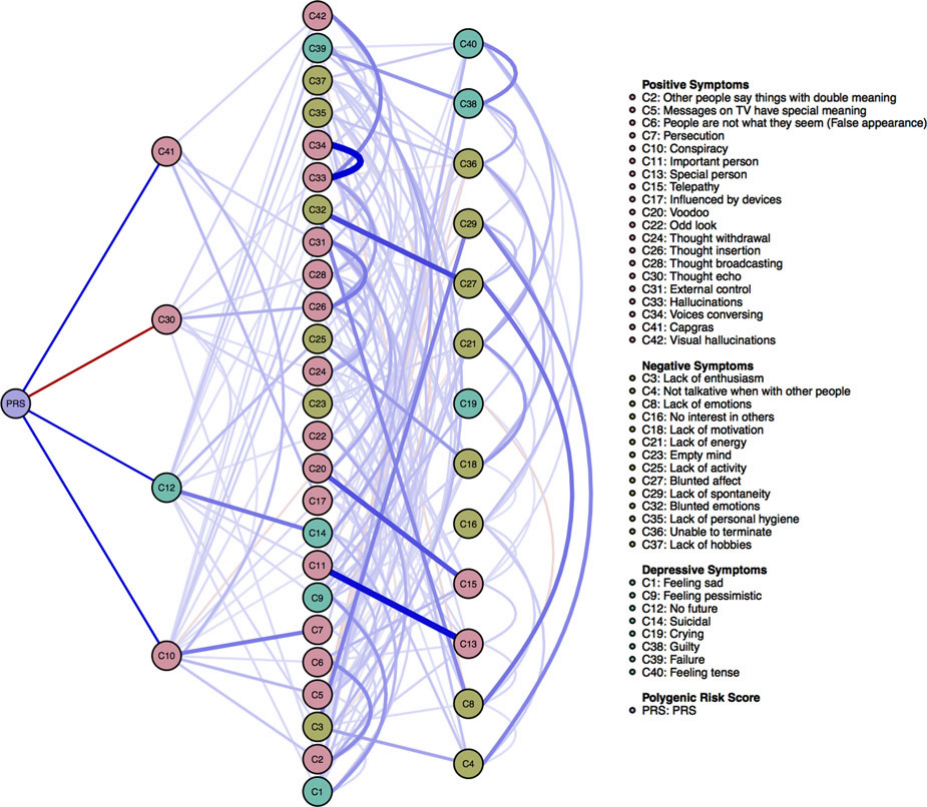
Predictive path diagram of the 42 CAPE (Konings *et al*., 2006) symptoms and the PRS for psychosis (*n* = 2180). A predictive path diagram is based on *shortest pathway analysis* (Brandes, 2008; Opsahl *et al*., 2010) and represents the first three levels of connectivity between variables. Specifically, here we visualize (1) the immediate nodes to which the PRS is connected; (2) the immediate nodes to which the nodes that are connected to the PRS are connected; and (3) the nodes to which the latter are connected. Blue (red) lines represent positive (negative) associations between variables and the wider and more saturated the edge, the stronger the association (Epskamp *et al*., 2012). Please note that since the focus of the paper was to investigate the relations between the PRS and symptoms, the edges between the PRS and symptoms have been manually un-faded (i.e. within the first level of connectivity), while the edges between the other nodes retained default plotting functions. Symptom groups are differentiated by color.

Second, the predictive path diagram (Fig. 2) highlights secondary levels of connectivity within the network – level two connections show strong relations between symptoms *C10* (conspiracy), *C7* (persecution) and *C41* (Capgras), as well as between symptoms *C12* (no future) and *C14* (suicidal). Level three connections display the strongest relations between symptoms *C33* (hallucinations) and *C34* (voices conversing), *C32* (blunted emotions) and *C27* (blunted affect), *C20* (voodoo) and *C15* (telepathy), and *C11* (important person) and *C13* (special person). Within this level, most symptoms were connected to most of the other symptoms. Notably, both in level two and three, most items that had strong links with other items were in fact linked to an item from the same dimension. Level three of connectivity was formed mostly of the positive symptoms, while level four of connectivity was formed mostly of the negative symptoms and the relations between these. Overall, the predictive path diagram shows that it becomes possible to reach any other symptom in the network from the PRS in no more than four steps. Nonetheless, these results should be interpreted with caution considering the large variability in edge weights and the presence of negative edges.

**Fig. 3.**
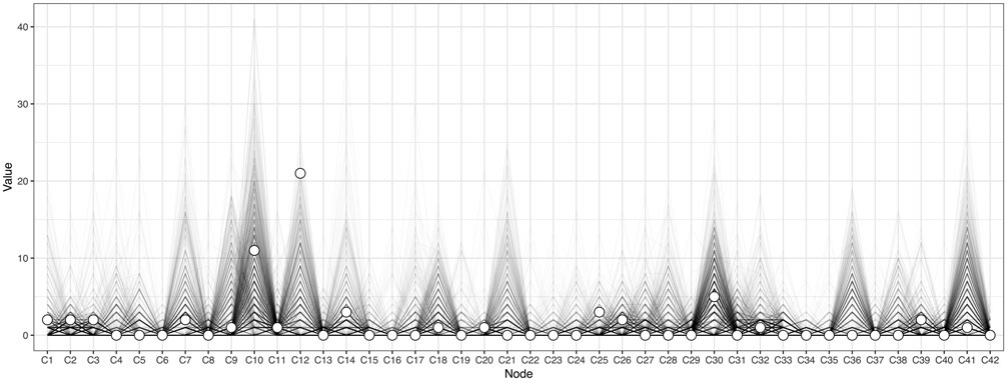
Node-specific predictive betweenness (i.e. how often a node lies on the pathways between two other nodes, of which one is always the PRS). The white dots represent the node-specific predictive betweenness in the current sample, while the black lines represent the variability of node-specific betweenness when using nonparametric bootstrapping over 1000 iterations.

Bootstrapping showed that the estimation of node-specific predictive betweenness (i.e. items that more often lie on the shortest pathways from PRS to other nodes; Fig. 3) was considerably less precise than that of other features of the network. In particular, Fig. 3 shows that several nodes featured high node-specific predictive betweenness in bootstrap samples but not in the centrality of the estimated network structure based on the sample, indicating that the shortest paths from PRS to other nodes can take various forms. A potentially important result that was robustly present across case-drop bootstraps (online Supplementary Fig. S4) is that the most central symptoms in the current sample in terms of node-specific predictive betweenness are symptoms *C12* (no future) and *C10* (conspiracy). Other central symptoms – even though to a much lesser degree and possibly as secondary items on the pathway between PRS and other nodes – were items *C25* (lack of activity), *C30* (thought echo), and *C39* (failure). Additionally, the nonparametric bootstrapped networks showed items *C9* (feeling pessimistic), *C21* (lack of energy), and *C36* (unable to terminate) as further potentially important nodes, even though these were not specifically captured as high node-specific predictive betweenness items in the current network structure (Fig. 3, black lines).

## Discussion

The current study provides, to the best of our knowledge, a first application of the network framework (Borsboom and Cramer, 2013) to the field of behavior genetics research. This allows for a novel outlook on the investigation of the relations between GWAS-based PRSs and symptoms of mental disorders, by focusing on the dependencies among variables.

Most links we identified in our data are links between the PRS and positive psychotic symptoms, especially symptoms related to notions of *conspiracy* and *paranoia*. This can be especially of interest as studies including risk factors in network models of psychosis (e.g. cannabis use, childhood trauma, urbanization) did not identify direct relations between any of these risk factors and positive or negative psychotic symptoms (Isvoranu *et al*., 2016*a*, 2016*b*). In addition, positive psychotic symptoms are generally found to have few connections to other nodes, especially nodes related to real life functioning (Galderisi *et al*., 2018). In light of this, a plausible hypothesis to put forward would be that different types of risk factors may lead to different pathways to the onset (and development) of psychotic disorders: genetic risk factors may have more of a main effect on the liability to develop positive psychotic symptomatology, while environmental risk factors may have more of a secondary effect on general psychopathology symptoms. This may partly explain why environmental factors are often shared between distinct mental disorder categories and is in line with previous findings which argued that symptoms can have different risk factors (Fried *et al*., 2014, 2015*a*, 2015*b*; Isvoranu *et al*., 2016*a*, 2016*b*).

To further investigate symptom associations at different levels of connectivity, the current study constructed for the first time a *predictive path diagram*. To a certain degree, the predictive path diagram aims to point out toward potential mediating items. Here, the second level of symptom–symptom interactions consisted of mostly positive psychotic symptoms, while the third level of symptom–symptom interactions had a predominance of negative psychotic symptoms. Most of the symptoms connected to other symptoms from the same domain, suggesting that node activation may first spread within the same domain. In addition, since most links were identified between the PRS and positive psychotic symptoms, the second level included most of the positive psychotic symptoms. The few negative symptoms identified at the second level of symptom–symptom interactions are items related to lack of enthusiasm, energy, and (social) activities. The only two positive psychotic symptoms identified at the third level of interaction are *telepathy* and *special person*. Depressive symptoms are shared between the two levels and generally have less strong relations within and between domains. It should be noted that the symptoms with the strongest relations between different levels of connectivity are highly similar in content to each other (e.g. strong relations between items *C10: Do you ever feel as if there is a conspiracy against you?* and *C7: Do you ever feel as if you are being persecuted in some way?* or between items *C32: Do you ever feel that your emotions are blunted?* and *C27: Do you ever feel that your feelings are lacking in intensity?*). This result may therefore be an artifact of the scale used to measure symptomatology, in which many of the items are contextually similar and therefore are likely highly correlated with each other. Further research using different measurement scales is warranted before drawing strong conclusions based on this methodology.

Finally, for the current study we developed a measure of *node-specific predictive betweenness*, which allows for the investigation of items that more often lie on the shortest pathway from PRS to other nodes in the network. We identified the items *conspiracy* and *no future* as central items and hypothesize that these may be important hubs in the network.

### Methodological notes

Several qualifications of the network methodology are in order here. First, the statistical approach of incorporating PRS in a network is suited to pick up direct links between the PRS and psychotic symptoms. As such, this methodology is best interpreted as incorporating the hypothesis that PRS contains genetic factors that increase the liability to develop certain symptoms (i.e. acts through main effects on the symptom thresholds in a network). This is because PRS is modeled as a predictive node from the causal background.

Second, predictive nodes from the causal background are of course unlikely to operate on the same timescale as symptoms in a symptom network. However, the nature of statistical control in a partial correlation network which includes PRS as a node is likely not that different from that of a network that exclusively contains symptoms. If, for instance, PRS was a common cause for two symptoms within a network structure (e.g. increased PRS leads to increased liability to develop symptoms *sadness* and *conspiracy*) but not included in the network (and thus not controlled for), a spurious edge between the two symptoms would be induced. If PRS was indeed linked to symptoms, such spurious associations would then be eliminated due to the inclusion of PRS as a node in the network. Of note, a node only induces spurious connections if it is a common effect in a network (Epskamp *et al*., 2018), but PRS cannot be a common effect, because it is not caused by symptoms. Therefore, to the extent that stable edges arise between PRS and symptomatology, such edges are consistent with the hypothesis that PRS impinges at the symptom level.

Third, it is widely known that genetic influences account for a small proportion of the explained variance in a mental disorder and even though this proportion is larger for psychosis, the effect is still modest (Greenwood *et al*., 2007). Naturally, symptoms tend to cluster together and have a stronger influence on each other, while variables from the external field of a disorder, such as environmental and genetic factors also better cluster together within their domain. Networks therefore often display weaker between-domain links (Isvoranu *et al*., 2016*b*; Santos *et al*., 2017), and may have missed between-domain links due to a lack of statistical power. In addition, recent studies showed that when using estimation techniques relying on the lasso regularization (Tibshirani, 1994) – which many prior network studies have been based on – specificity can be lower than expected in dense networks with many small edges, leading to an increase in false positives (Williams and Rast, 2018). To this end, we chose a different and more conservative estimation technique here based on unregularized model search, as recently recommended by Epskamp (2018). For low-dimensional settings with high sample size, simulation studies using this technique have shown good sensitivity as well as high specificity. We compared this method with the classic lasso regularization method, as well as with a method based on thresholded partial correlations. The unregularized model search performed best in terms of goodness-of-fit measures. In addition, the edges from PRS to *conspiracy* and *no future* were identified by two of the three methods, while the edges from PRS to *thought echo* and *capgras* were identified by all three methods (see online Supplementary Fig. S5 and Table S1). Overall, based on these results, the effects identified in this study are unlikely to be simply a result of noise and may hold etiological significance. In addition, bootstrap analyses (see online Supplementary Fig. S2) show the edges from PRS to *C41: capgras* and *C30: thought echo* are the most robust (i.e. showing up in 67% of the bootstraps and 75% of the bootstraps respectively). Notably, all edges from the PRS are identified in at least 50% of the bootstraps. Since the network was not constructed using *p* values but was based on a model fitting approach, which has been shown to be highly effective in type-I error control, multiple testing should not pose as an issue here.

### Limitations

The findings of this study need to be considered in light of several limitations. First, a PRS itself can be considered an important limitation. The signal between a PRS and symptoms is generally weak (Greenwood *et al*., 2007), also resulting in weak network connections, which are not overly consistent across different estimation techniques. In addition, by adding up SNPs to construct the PRS we may obscure details in the same way in which adding up items into sum-scores may obscure the different symptoms – investigating individual SNPs in relation to symptoms may therefore be a promising new avenue. It is further possible that the predictive power of the PRS also becomes weaker in broader mixed samples across the spectrum. The sample analyzed in this paper is a heterogeneous sample, which includes controls, siblings, parents and patients; in addition, as the CAPE is not designed for measuring symptomatology in patients, ceiling effects may occur when applying the CAPE to the patient population. However, given that the strong phenotypic manifestation of the PRS can often be identified in healthy relatives of patients (van Os *et al*., 2017), we chose to include the whole sample rather than focus on one group only. Ideally it would be possible to carry out analyses and direct network comparisons between different population groups; this was not possible in the current study due to limited data. Second, it is difficult to investigate to what degree any PRS effect would be reducible to group membership (i.e. patients have much more psychopathology and higher PRS scores). Including a *group membership* variable in a network would be problematic (Fried and Cramer, 2017) due to the categorical nature of the variable and therefore investigating to what extent there would be overlap between the two variables was not possible. A future extension for network modeling of genetic data may be modeling the multilevel structure of the data. Third, the negative association between PRS and thought echo is a puzzling finding, which could be related to some form of measurement bias or rarity, but could also be a true effect given a fixed level of other symptoms. Further investigation showed that, even though the expectation would be that PRS would positively correlate with all other variables, PRS and though echo were actually also weakly marginally negatively correlated (*r* = −0.004), thus ruling out that the result is paradoxical (i.e. a marginal positive correlation was not made negative upon conditioning). One possible alternative explanation to the stronger negative association in the network is that it may be due to the presence of a collider in the data (i.e. both PRS and thought echo cause a third variable in the dataset; Pearl, 2000). Another explanation may be that this is a spurious effect, resulting from the violation of the normality assumption (i.e. the variable ‘thought echo’ is relatively skewed to the left). Future research should focus on if the link between PRS and thought echo is a genuine negative effect that can be replicated. Finally, the current analyses were based on cross-sectional data and conclusions regarding direction or causality should be drawn with caution. In addition, it may be argued that results from cross-sectional networks may not be generalizable to within-person dynamics (Bos *et al*., 2017). It should however be noted that longitudinally investigating variables from the external field of a network, such as environmental or genetic risk factors, is methodologically infeasible as these background variables may not change themselves; in addition, as we argued in this manuscript, if such variables act to facilitate the ease with which nodes in the network are activated, the current analysis may in fact be more informative than a within-subject analysis of dynamically changing factors would by itself be. Future research could expand on our analyses by investigating whether PRS might further have an effect on the interactions between symptoms, using *network moderation* techniques (Haslbeck *et al*., 2018) or by relating PRS to parameters of networks that govern dynamic changes in symptomatology.

## Conclusion

Overall, the results of our study indicate that investigating pathways within a network structure through which genetic components may affect the liability to develop a mental disorder may be promising and may open new research avenues. Here we have taken a first step toward introducing this methodology. Further investigating how (and whether) other PRSs are related to (other) symptoms, as well as whether we can identify certain genetic segments for specific symptoms could lead to biological informative structures. The current study is exploratory in nature; in light of this, we discuss methodological considerations and limitations of the approach and argue further research replicating these results is essential. Ultimately, we aim to provide a first means toward bringing together the field of behavior genetics and the network framework, and provide preliminary results for symptom-specific biological pathways to psychosis.

## Note

1 The estimation procedure first runs the glasso algorithm (Friedman *et al.*, 2008) for 100 different tuning parameters to obtain 100 different network structures (ranging from sparse networks with few edges to dense networks with many edges). Next, the algorithm refits all those networks without regularization and selects the model that minimizes the Bayesian information criterion (BIC). Subsequently, the algorithm adds and removes edges until BIC can no longer be improved. It has been shown that BIC selection of GGMs selects the true model as *N* grows to infinity (Foygel & Drton, 2011). In addition, the stepwise procedure ensures that no single edge can be removed or added in the final model to improve fit.

2 This is because the software used for constructing the networks automatically fades edges with a smaller effect – an effect between a variable from the external field of a symptom (such as an environmental variable or a genetic risk score; Borsboom, 2017) will likely almost always be smaller than the effect of the variables within the network (e.g. the relations between the symptoms themselves).

3 Of note, when running the same analysis within the subsample of patients and relatives only, the relations between the PRS and *C10* (Conspiracy), and *C41* (Capgras) are retained and no other edges emerge. It is likely that edges between PRS and *C12* (No future) and *C30* (Thought echo) do not withhold due to a loss in power.

## Supplementary material

For supplementary material accompanying this paper visit http://dx.doi.org/10.1017S003329171900045X. click here to view supplementary material
