# Supplementary material for: Toward incorporating genetic risk scores into symptom networks of psychosis
Source: Psychol Med. 2019 Mar 14;50(4):636–43. doi: 10.1017/S003329171900045X (PMC7093319; doi:10.1017/S003329171900045X)
Supplement: Supplementary file 1 [file S003329171900045Xsup001.pdf]

Supplementary Online Content

Isvoranu, AM, Guloksuz S, Epskamp S, van Os J, Borsboom D, GROUP (2018) Towards Incorporating Genetic Risk Scores into Symptom Networks of Psychosis

Appendix S1. Genotyping, imputation and PRS

Figure S1. Schizophrenia PRS explained variance of schizophrenia outcome in GROUP data

Appendix S2. Accuracy and stability checks

Figure S2. Bootstrap results for edge-weights between PRS and all other nodes, based on 1,000 nonparametric bootstrap samples

Figure S3. Bootstrap results for all edge-weights, based on 1,000 nonparametric bootstrap samples

Figure S4. Average case-drop bootstraps of node-specific betwenneess, based on 1,000 iterations

Figure S5. Comparison of edge presence and strength across different estimation techniques

Table S1. Fit comparison across different estimation methods

Figure S6. Zero-order correlation network (with Bonferroni-corrected significance thresholding)

This supplementary material has been provided by the authors to give readers additional information about their work.

Appendix S1. Genotyping, imputation and PRS

Genotype data for 2,812 individuals was generated on a customized Illumina, IPMCN array with 570,038 SNPs. This chip contains ~250k common SNPs, 250K Exome chip variants (rare, exomic, nonsynonymous, MAF < 1%), and ~50K psychiatric-related variants. Quality control procedures were performed using PLINK v1.9 (Purcell *et al.*, 2007). SNPs and samples with call rates below 95% and 98%, respectively, were removed. A strict SNP QC only for subsequent sample quality control steps was conducted. This involved a minor allele frequency (MAF) threshold > 10% and a Hardy-Weinberg equilibrium (HWE) p-value > 1e-05, followed by linkage disequilibrium (LD) based SNP pruning ($R^2 < 0.2$). This resulted in ~58K SNPs to assess sex errors, heterozygosity (F < 3 standard deviation (SD)), homozygosity (F>3SD) and relatedness by pairwise identity by descent (IBD) values. Duplicate samples (pihat > 0.8) were removed and remaining pairs were manually checked since this dataset contains family members. After removing failing samples, a regular SNP QC was performed (SNP call rate > 98%, HWE p > 1e-06, MAF > 1%). After MDS clustering with Hapmap Phase 3 individuals to check ethnicity, samples that deviated more than 3 standard deviations from our dataset were removed (n = 91). In addition, the first 20 genetic PCs of passed quality controlled samples were generated using the strict SNP QC list by EIGENSTRAT (Price *et al.* 2006). Next, strand ambiguous SNPs and duplicate SNPs were removed. Mendelian errors were set to missing followed by another missingness check (2% threshold) for samples (n = 8) and SNPs, and SNPs with a differential missingness between cases and controls were removed. In total, 2,505 individuals and 275,021 SNPs passed these abovementioned QC steps.

SNPs were imputed on the Michigan server (Das *et al.* 2016) using the HRC r1.1 2016 reference panel with European samples after phasing with Eagle v2.3. Post-imputation QC involved removing SNPs with an Rsq info score < 0.3, with a MAF < 0.01, SNPs that had a

discordant MAF compared to the reference panel, and strand ambiguous AT/CG SNPs and multi-allelic SNPs.

PRS calculations

Polygenic risk scores (PRS) for 2,505 samples were calculated using schizophrenia-associated alleles and effect sizes reported in the GWAS summary statistics from the Psychiatric genetics consortium (PGC) 2014 (Ripke *et al*. 2014), excluding Dutch subjects. Overlapping SNPs between the PGC GWAS (training dataset), 1000 reference Genome (reference dataset), and our dataset (target dataset) were selected. Then 1) insertion or deletion, ambiguous SNPs; 2) SNPs with minor allele frequency (MAF) < 0.01 and SNPs with imputation quality (R^2) < 0.8 in both training dataset and target dataset; 3) SNPs located in complex-LD regions (Price *et al.* 2008), were excluded, leaving 2,950,238 SNPs. These SNPs were clumped in two rounds using PLINK; round 1 with the default parameters (physical distance threshold 250kb and LD threshold (R^2) < 0.5; round 2 with a physical distance threshold of 5,000kb and LD threshold (R^2) < 0.2; resulting 194,665 SNPs for PRS calculation. Odds ratios for autosomal SNPs reported in the schizophrenia summary statistics were log-converted to beta values. PRS were calculated using PLINK's score function for 12 schizophrenia GWAS p-value thresholds: 5×10^{-8}, 5×10^{-7}, 5×10^{-6}, 5×10^{-5}, 5×10^{-4}, 5×10^{-3}, 0.05, 0.1, 0.2, 0.3, 0.4 and 0.5. For the current analyses, guided by the PGC results, PRS at p-value threshold of 0.05 was used to achieve a balance between the number of false-positive and true-positive risk alleles.

PRS analyses

From 2,505 individuals, we selected 706 SCZ cases and 368 unrelated healthy controls into the binomial logistic regression model. The model equation is

$$\log\left(\mathrm{SCZ}_{\mathrm{case}_{\mathrm{control}_{\mathrm{status}}}}\right) \sim \mathrm{age} + \mathrm{sex} + \mathrm{PC1} + \mathrm{PC2} + \mathrm{PC3} + \mathrm{PRS}.$$

The explained variance is represented as Nagelkerke r2, conducted by the 'descr' (Aquino *et al.* 2009) package in R (R Development Core Team, 2015). For details see Figure S1 below.

Figure S1. Schizophrenia PRS explained variance of schizophrenia outcome in GROUP (Korver *et al.* 2012) data

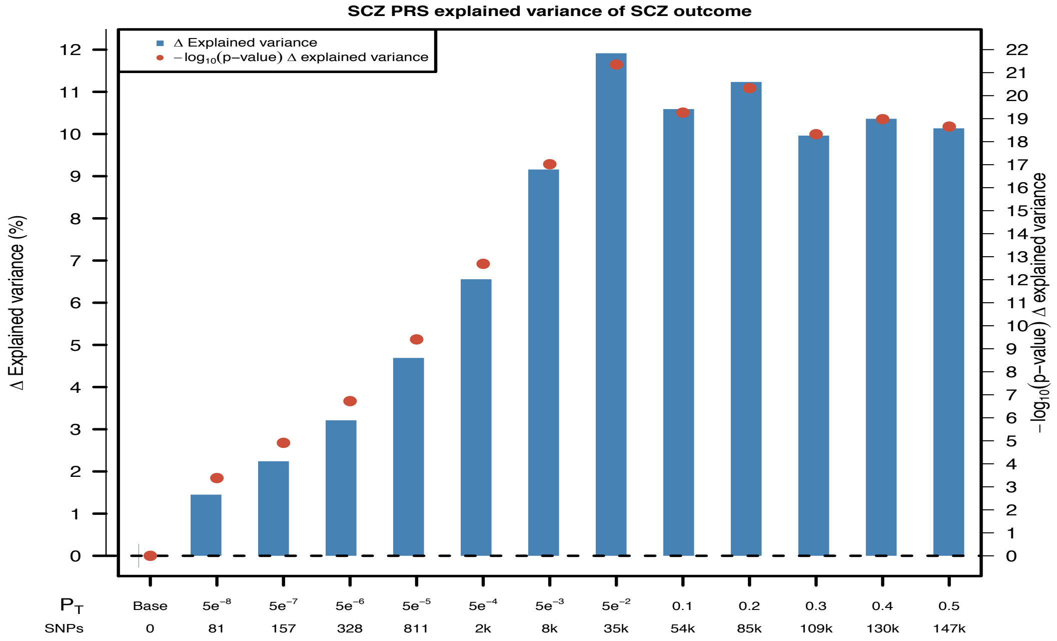

P-value thresholds (P_T) for SZ SNPs are shown on the x axis, where the number of SNPs increases with a more lenient P_T. Δ Explained variances (Nagelkerke R^2, shows as a %) of a generalized linear model including SZ-based PRS versus a baseline model without polygenic scores (blue bars) are shown for each P_T. $-\mathrm{Log}_{10}$ P-values of Δ explained variance per P_T (red dots) represent P-values from the binomial logistic regression.

Appendix S2. Accuracy and stability check for the estimated network model

In order to check whether the estimated network connections and centrality measure were accurate, we carried out bootstrap stability checks, using the R-package *bootnet* (Epskamp *et al.* 2017). Figure S2 below shows bootstrap results for edge-weights between PRS and all other nodes, Figure S3 below shows bootstrap results for all edge weights, and Figure S4 shows the average case-drop bootstraps of node-specific betweenness. An extensive interpretation of each result is included in the respective figure captions.

Figure S2. Bootstrap results for edge-weights between PRS and all other nodes, based on 1,000 nonparametric bootstrap samples

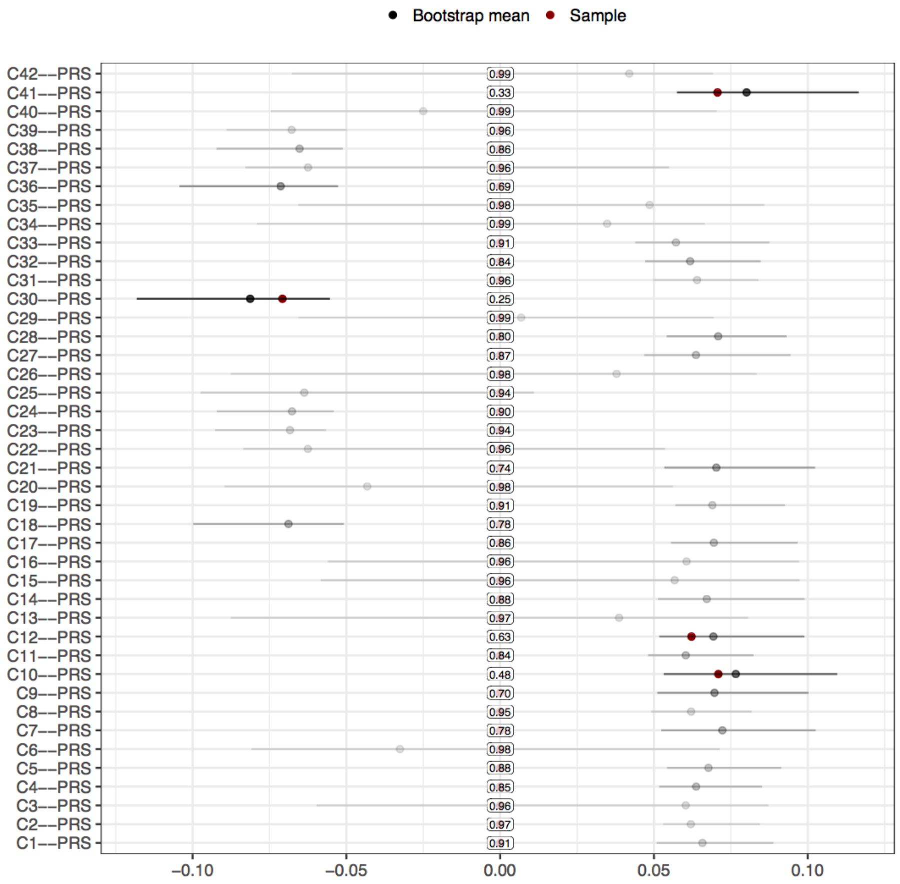

Each horizonal line within the plot represents the 95% quantile range of the parameter values *when the parameter was included in the model*. The red dots indicate the sample values for the analyzed data, while the grey dots indicate the bootstrap mean values. The values displayed in the center of the plot show the percentage of bootstraps in which an edge was zero; in addition, the more faded the line, the higher the percentage of bootstraps in which an edge was zero. The sample values lie within the bootstrapped confidence intervals and the bootstrap confidence intervals are relatively small, thus indicating accurate estimations. The edges from PRS to C42 and C30 are the most robust (i.e., showing up in 67% of the bootstraps and 75% of the bootstraps respectively). Notably, all edges are identified in at least 50% of the bootstraps.

Figure S3. Bootstrap results for all edge-weights based on 1,000 nonparametric bootstrap samples

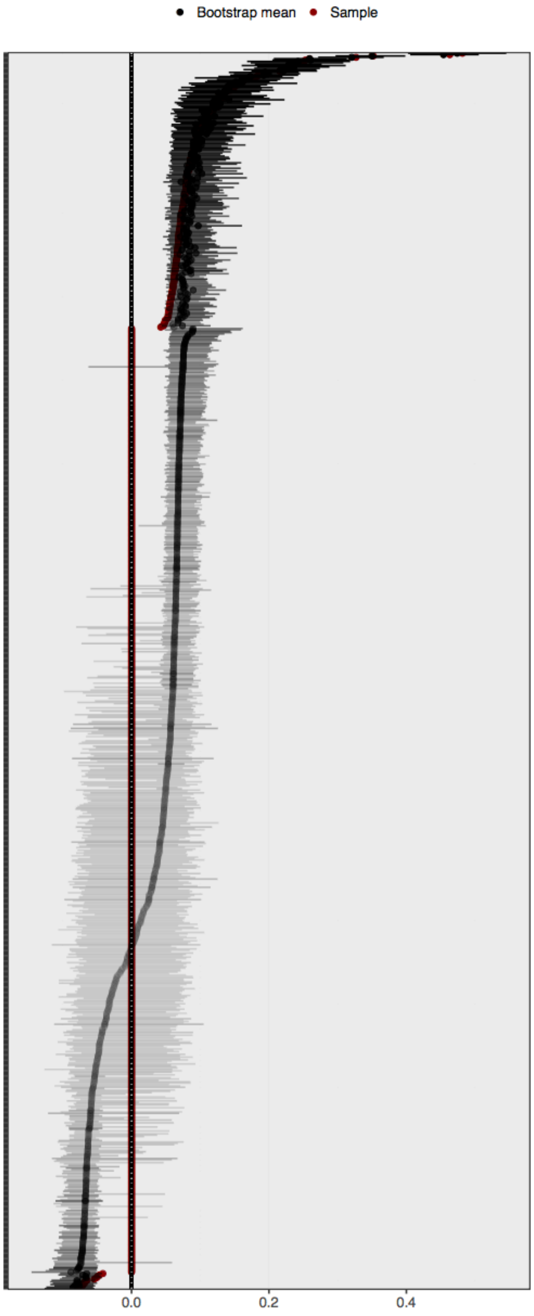

Each horizontal line indicates the 95% quantile range of the parameter values *when the parameter was included in the model*. The transparency of each line indicates the proportion of times the edge was included in the model (more transparent lines indicate edges that were included less often). The red dots within the plot indicate the sample values for the analyzed data, while the grey areas indicate bootstrapped confidence intervals.

Figure S4. Average case-drop bootstraps of node-specific betweenness, based on 1,000 iterations

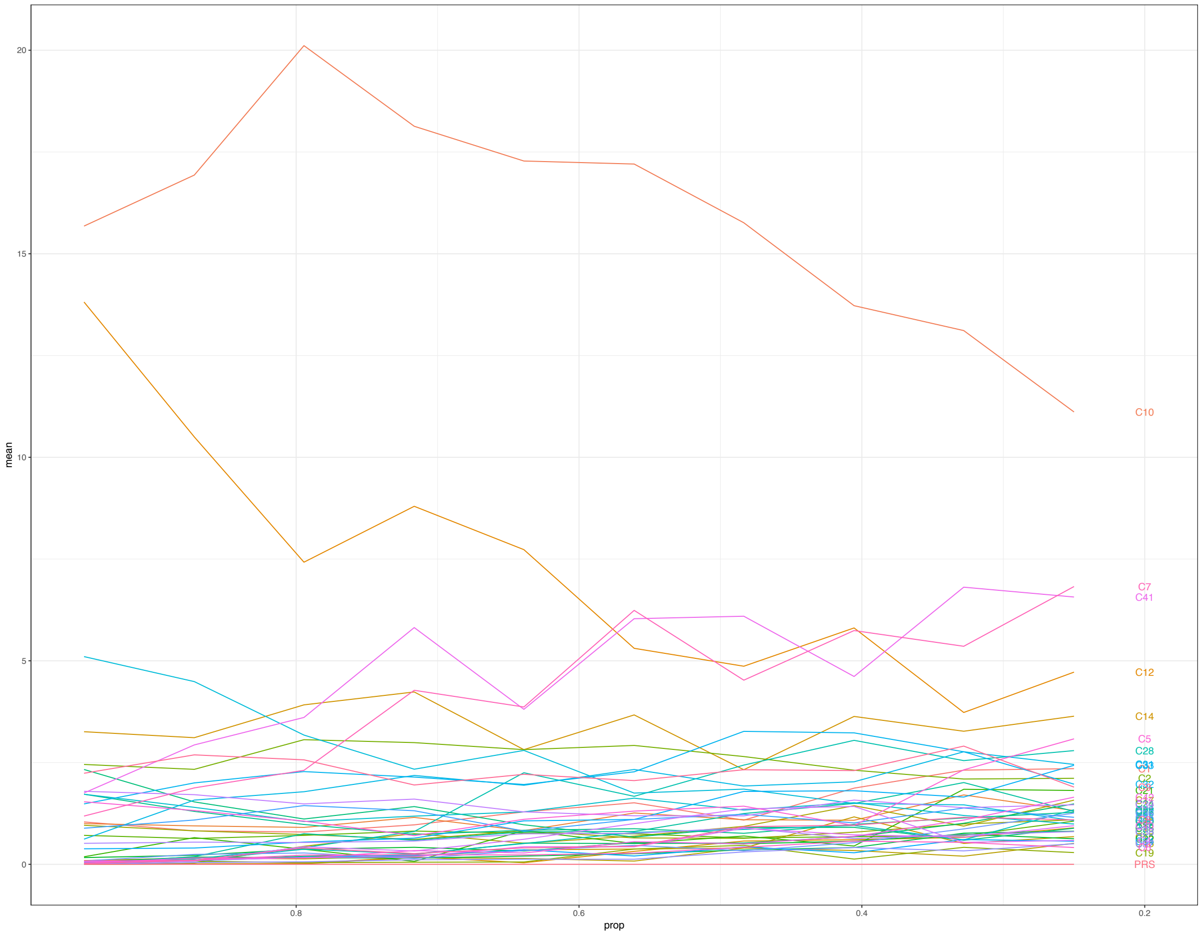

The lines represent how node-specific betweenness (i.e., how often a node lies on the pathways between two other nodes, of which one is always the PRS) changes for each variable when dropping different proportions of the data. Overall the stability is not very reliable, but nodes C10 and C12 retain relatively high node-specific betweenness across case-drops.

Figure S5. Comparison of edge presence and strength across different estimation techniques

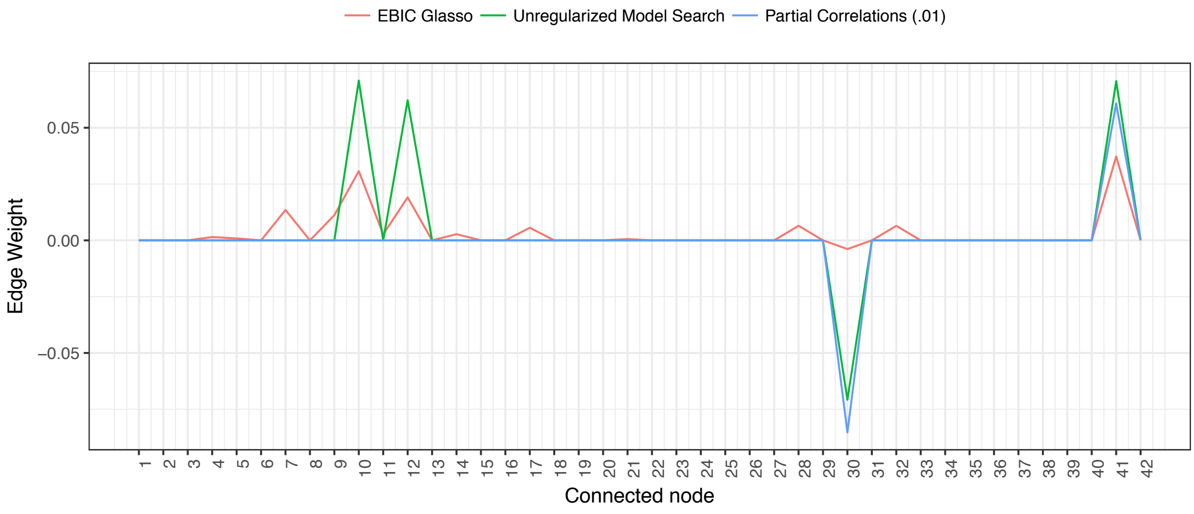

The lines indicate the presence and strength of the edges identified between the PRS and other nodes in the network when using different estimation techniques (i.e., the EBICglasso with a tuning parameter of .5, unregularized method search, and partial correlations thresholded at alpha .01). The edges between PRS and nodes 30 and 42 are identified by all estimation methods, while the edges between PRS and nodes 10 and 12 by two out of three estimation methods.

Table S1. Fit comparison across different estimation methods (Epskamp *et al.* 2017)

	AIC	BIC	EBIC	RMSEA	CFI
Unregularized Model Search	231618.6	233811.0	236178.6	0.015	0.990
EBICglasso	231845.0	234216.5	240627.6	0.018	0.989
Partial correlations alpha .01	232935.2	233811.0	236178.6	0.032	0.951

Figure S6. Zero-order correlation network (with Bonferroni-corrected significance thresholding)

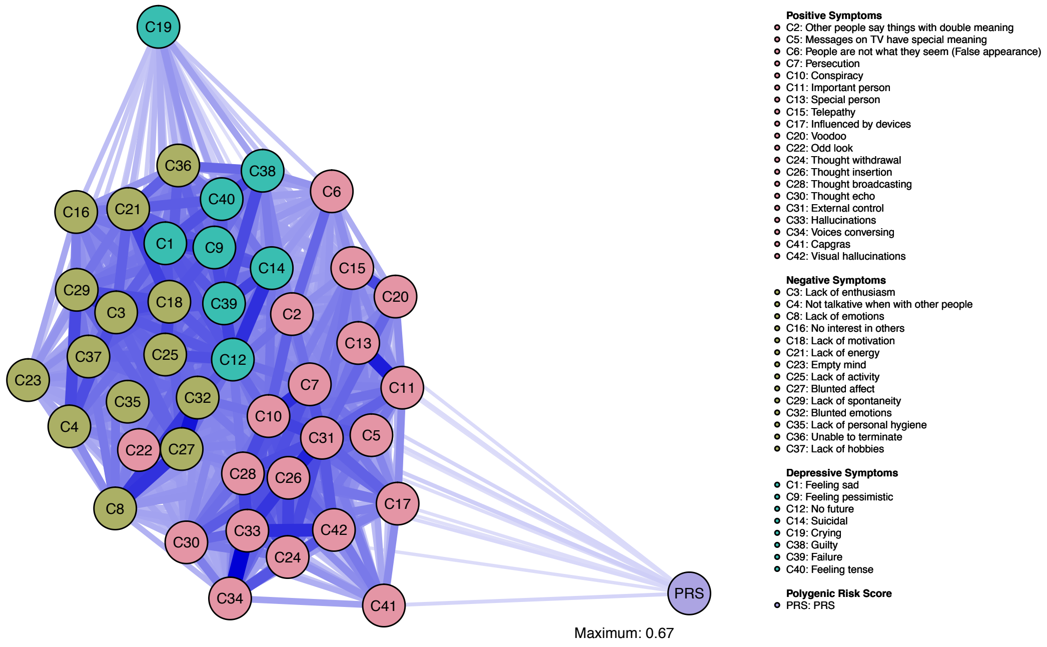

Network of the 42 CAPE (Konings et al. 2006) symptoms and the PRS for psychosis (n = 2,180). Blue lines represent positive associations (i.e., here zero-order correlations with Bonferroni-corrected significance thresholding) between variables and the wider and more saturated the edge, the stronger the association (Epskamp et al. 2012). Symptom groups are differentiated by color. PRS is positively associated with items C5, C7, C9, C10, C11, C12, C14, C17, C27, C28, C31, C32, C33, C41.

References

Aquino J, Enzmann D, Schwartz M, and Jain N (2009) descr: Descriptive statistics. R package. https://cran.r-project.org/package=descr.

Das S, Forer L, Schönherr S, Sidore C, Locke AE, Kwong A, Vrieze SI, et al. (2016). Next-Generation Genotype Imputation Service and Methods. *Nature Genetics* **48**, 1284.

Epskamp S, Borsboom D, and Fried EI (2018). Estimating Psychological Networks and Their Accuracy: A tutorial paper. *Behavior Research Methods* **50**, 195-212.

Epskamp S, Cramer AOJ, Waldorp LL, Schmittmann VD, and Borsboom D (2012). Qgraph : Network Visualizations of Relationships in Psychometric Data. *Journal of Statistical Software* **48**: 1–18. http://www.jstatsoft.org/v48/i04.

Epskamp S, Rhemtulla M, and Borsboom D (2017) Generalized Network Psychometrics: Combining Network and Latent Variable Models, *Psychometrika* **82**, 904-927.

Konings M, Bak M, Hanssen M, van Os J, and Krabbendam L (2006) Validity and Reliability of the CAPE: A Self-Report Instrument for the Measurement of Psychotic Experiences in the General Population. *Acta Psychiatrica Scandinavica* **114**: 55–61.

Korver N, Quee PJ, Boos HB, Simons CJ, de Haan L (2012) Genetic Risk and Outcome of Psychosis (GROUP), a Multi-Site Longitudinal Cohort Study Focused on Gene-Environment Interaction: Objectives, Sample Characteristics, Recruitment and Assessment Methods. *International journal of methods in psychiatric research* **21**, 205-221.

Price, AL, Patterson NJ, Plenge RM, Weinblatt ME, Shadick NA, and Reich D (2006) Principal components analysis corrects for stratification in genome-wide association studies. *Nature Genetics* **38**, 904.

Price AL, Weale ME, Patterson N, Myers SR, Need AC, Shianna KV, Dongliang G, *et al.* (2008) Long-Range LD Can Confound Genome Scans in Admixed Populations,

American Journal of Human Genetics **83**, 132-135.

Purcell S, Neale B, Todd-Brown K, Thomas L, Ferreira MAR, Bender D, Maller J, *et al.* (2007) PLINK: A Tool Set for Whole-Genome Association and Population-Based Linkage Analyses. *The American Journal of Human Genetics* **81**, 559-575.

R Development Core Team (2014) R: A Language and Environment for Statistical Computing. R Foundation for Statistical Computing, Vienna, Austria.

Ripke S, Neale BM, Corvin A, Walters JTR, Farh KH, Holmans PA, Lee P *et al.* (2014). Biological Insights From 108 Schizophrenia-Associated Genetic Loci. *Nature* **511**, 421–27.
